# *HER2*基因状态与晚期肺腺癌患者一线培美曲塞联合铂类化疗疗效的关系

**DOI:** 10.3779/j.issn.1009-3419.2019.03.03

**Published:** 2019-03-20

**Authors:** 盼华 李, 班班 李, 云舒 史, 凤鸣 张, 淑景 申, 醒亚 李

**Affiliations:** 1 450052 郑州，郑州大学第一附属医院肿瘤科 Department of Medical Oncology, the First Affiliated Hospital of Zhengzhou University, Zhengzhou 450052, China; 2 450052 郑州，郑州大学第一附属医院放疗科 Department of Tumor Radiotherapy, the First Affiliated Hospital of Zhengzhou University, Zhengzhou 450052, China

**Keywords:** 肺肿瘤, HER2, 培美曲塞, 一线化疗, Lung neoplasms, HER2, Pemetrexed, First-line chemotherapy

## Abstract

**背景与目的:**

人类表皮生长因子受体2（human epidermal growth factor receptor 2, *HER2*）突变是非小细胞肺癌（non-small cell lung cancer, NSCLC）的分子标记物之一，研究显示培美曲塞在*HER2*突变NSCLC中的疗效存在争议。本研究拟探讨培美曲塞联合铂类化疗在*HER2*突变和HER2野生型肺腺癌患者中的疗效。

**方法:**

回顾性分析在郑州大学第一附属医院经组织病理学证实的106例EGFR、ALK、ROS-1、KRAS、BRAF、RET、MET均为阴性的晚期肺腺癌患者的临床资料。分析*HER2*基因状态、临床特征、化疗疗效及无进展生存期（progression-free survival, PFS）之间的关系。

**结果:**

106例患者均进行了*HER2*基因检测，*HER2*突变32例（30.2%），未发生突变74例（69.8%）。*HER2*突变在年轻、未吸烟、女性患者中多见。所有患者均接受一线培美曲塞联合铂类的化疗，*HER2*突变肺腺癌患者的客观缓解率（objective response rate, ORR）和疾病控制率（disease control rate, DCR）均高于HER2野生型患者（40.6% *vs* 14.9%, *χ*^2^=8.464, *P*=0.004; 93.8% *vs* 68.9%, *χ*^2^=6.327, *P*=0.012），差异有统计学意义。单因素分析显示：PFS与有无脑转移、有无维持化疗和*HER2*基因状态相关（*P* < 0.05），而与年龄、性别、是否有吸烟史、是否寡转移、有无肝转移及铂的种类无关（*P* > 0.05）。*Cox*多因素分析显示：*HER2*突变是PFS的独立正性预后因素（*P*=0.038）。

**结论:**

*HER2*突变相比*HER2*野生型肺腺癌患者一线应用培美曲塞联合铂类的化疗有更大的临床获益。

## Abstract (en)

**Background and objective:**

Human epidermal growth factor receptor 2 (*HER2*) is one of the driver genes of non-small cell lung cancer (NSCLC). Several studies have shown that the efficacy of pemetrexed in HER2-mutant NSCLC is controversial. The aim of this study is to investigate the efficacy of pemetrexed combined with platinum chemotherapy in patients with *HER2*-mutant and HER2 wild-type lung adenocarcinoma.

**Methods:**

The clinical data of 106 cases of EGFR, ALK, ROS-1, KRAS, BRAF, RET and MET-negative patients with advanced lung adenocarcinoma patients who diagnosed by histopathology in the First Affiliated Hospital of Zhengzhou University were retrospectively reviewed. The relationships between *HER2* gene status, clinical characteristics and response and progression-free survival (PFS) were analyzed.

**Results:**

All of the 106 patients' HER2 status were determined. *HER2* mutations occurred in 32 cases (30.2%), no mutations in 74 cases (69.8%). *HER2* mutations were common in young, non-smoking and female patients. All patients received first-line pemetrexed and platinum-based chemotherapy. The objective response rate (ORR) and disease control rate (DCR) of patients with *HER2*-mutant lung adenocarcinoma were significantly higher than those without *HER2* mutations (40.6% *vs* 14.9%, *χ*^2^=8.464, *P*=0.004; 93.8% *vs* 68.9%, *χ*^2^=6.327, *P*=0.012), and the difference was statistically significant. According to univariate analysis, the PFS was significantly associated with the brain metastases, maintenance chemotherapy and *HER2* gene status (*P* < 0.05), but not with age, gender, smoking history, oligometastases, liver metastases and type of platinum (*P* > 0.05). *Cox* multivariate analysis indicated that *HER2* mutation was an independent positive prognostic factor of PFS (*P*=0.038).

**Conclusion:**

*HER2*-mutant lung adenocarcinoma patients with first-line pemetrexed combined with platinum chemotherapy have greater clinical benefit than HER2 wild-type patients.

肺癌是发病率和死亡率最高的恶性肿瘤，全球每年约有180万的新发肺癌患者，其中非小细胞肺癌（non-small cell lung cancer, NSCLC）约占肺癌的85%^[1, 2]^。NSCLC进一步分型为腺癌、鳞癌和大细胞癌，早期NSCLC患者可选择手术治疗，但大部分患者确诊时已属晚期，错失手术机会，因此化疗和分子靶向治疗是目前晚期NSCLC患者的主要治疗选择。

人类表皮生长因子受体2（human epidermal growth factor receptor 2, HER2）属于HER蛋白家族之一，HER蛋白家族其他成员包括HER1、HER3和HER4^[3]^。*HER2*基因异常包括HER2激酶结构域突变、蛋白过表达和拷贝数增加。研究^[4]^表明，与HER2蛋白过表达和拷贝数增加相比，*HER2*突变是NSCLC的分子生物标记物之一。14年前Stephens等^[5]^首次报道了肺癌HER2激酶结构域突变，但*HER2*突变在NSCLC的预测意义及*HER2*突变与化疗疗效之间的关系存在争议。一项国内回顾性研究^[6]^显示，一线以培美曲塞为基础的化疗在晚期*HER2*突变NSCLC患者的无进展生存期（progression-free survival, PFS）劣于*ALK*/ *ROS1*重排的患者。本研究旨在进一步探讨在晚期肺腺癌患者中*HER2*基因状态和一线培美曲塞联合铂类化疗疗效及PFS之间的关系，为临床提供指导。

## 1 资料和方法

### 1.1 病例选择

入组标准：①根据第七版肺癌肿瘤-淋巴结-转移（tumor-node-metastasis, TNM）分期标准证实的EGFR、ALK、ROS-1、KRAS、BRAF、RET、MET均为阴性的Ⅳ期肺腺癌患者，初治选用培美曲塞联合铂类的一线双药化疗；②化疗期间未接受同步或序贯放疗或其他抗肿瘤治疗；③根据实体瘤疗效评价标准（Response Evaluation Criteria in Solid Tumors, RECIST）1.1至少有1个可评价病灶，化疗期间至少2个周期进行一次影像学评估[胸加上腹部计算机断层扫描（computed tomography, CT）加或不加头颅磁共振成像（magnetic resonance imaging, MRI）]。

### 1.2 *HER2*基因检测方法

使用Sanger测序或二代测序（next generation sequencing, NGS）方法确认*HER2*基因突变状态。

### 1.3 疗效评价与随访

应用化疗2个周期后评价，疗效根据RECIST 1.1标准判定，分为完全缓解（complete response, CR）、部分缓解（partial response, PR）、疾病稳定（stable disease, SD）和疾病进展（progressive disease, PD）。客观缓解率（objective response rate, ORR）=（CR+PR）/（CR+PR+SD+PD）×100%；疾病控制率（disease control rate, DCR）=（CR+PR+SD）/（CR+PR+SD+PD）×100%。获得CR或PR的患者4周或以后确认。PFS定义为从化疗第一天至首次记录的疾病进展或死亡时间。采用电话和门诊方式进行随访，随访截止至2018年10月31日。

### 1.4 统计学方法

采用GraphPad Prism 7.0软件分析。分析*HER2*基因状态、临床特征与化疗疗效之间的关系采用卡方检验或*Fisher*精确检验。生存分析采用*Kaplan-Meier*法进行分析并进行*Log-rank*检验，采用*Cox*回归进行PFS的多因素分析。所有统计学检验均为双侧概率检验，以*P* < 0.05为差异有统计学意义。

## 2 结果

### 2.1 一般资料

回顾性分析2016年1月-2018年8月在郑州大学第一附属医院经组织学或细胞学确诊的125例EGFR、ALK、ROS-1、KRAS、BRAF、RET、MET均为阴性的Ⅳ期肺腺癌患者的临床资料。所有患者均进行了*HER2*基因检测。寡转移定义为治疗前转移灶数≤5个。培美曲塞均为国产用药，按500 mg/m^2^的剂量给予，联合铂类药物均为国产用药，包括顺铂、卡铂、奈达铂，分别按75 mg/m^2^、AUC 5×（GFR+25）mg和75 mg/m^2^的剂量给予，双药化疗4个-6个周期，每21天为1个周期。若无疾病进展，继续采用培美曲塞维持治疗，每28天为1个周期。为减少药物不良反应，所有患者接受叶酸、维生素B_12_和地塞米松预处理。排除随访丢失的患者，106例患者纳入此研究，其中男性63例，女性43例，年龄31岁-78岁，中位年龄为59岁；一线化疗中位化疗周期数为5个（1个-14个）；其中35例患者接受了培美曲塞维持治疗，维持周期数为1个-8个。

### 2.2 *HER2*基因状态与临床病理特征之间的关系

*HER2*突变患者32例（30.2%），年龄31岁-71岁，中位年龄为55岁；HER2野生型患者74例（69.8%），年龄34岁-78岁，中位年龄为60岁。*HER2*突变状态与年龄、性别、吸烟史相关，与是否寡转移无关。*HER2*突变在 < 65岁、女性、不吸烟的患者中更多见（表 1）。

**1:** *HER2*基因状态与临床特征的关系 Relationships between *HER2* gene status and clinical characteristics

Characteristic	*HER2*-mutant (*n*=32)	*HER2* wild-type (*n*=74)	*χ*^2^	*P*
Age (yr)			5.141	0.023
< 65	27	46		
≥65	5	28		
Gender			15.100	< 0.001
Male	10	53		
Female	22	21		
Smoking status			11.340	< 0.001
Ever	6	40		
Never	26	34		
Oligometastases			0.112	0.738
Yes	6	16		
No	26	58		
Efficacy evaluation				
ORR (%)	40.6	14.9	8.464	0.004
DCR (%)	93.8	68.9	6.327	0.012
ORR: objective response rate; DCR: disease control rate.				

### 2.3 *HER2*基因状态与化疗疗效之间的关系

106例患者均有可评价病灶。其中CR 0例（0%），PR 24例（22.6%），SD 57例（53.8%），PD 25例（23.6%），ORR为22.6%，DCR为76.4%。*HER2*突变肺腺癌患者的ORR和DCR均高于HER2野生型患者（40.6% *vs* 14.9%, *χ*^2^=8.464, *P*=0.004; 93.8% *vs* 68.9%, *χ*^2^=6.327, *P*=0.012），差异有统计学意义（表 1）。

### 2.4 *HER2*突变亚型与化疗疗效之间的关系

32例*HER2*突变肺腺癌患者的具体突变亚型包括：p. A775_G776insYVMA（*n*=17）；p. M774_A775insAYVM（*n*=7）；p. G776delinsVC（*n*=4）；V659E（*n*=1）；p. P780_Y781insGSP（*n*=1）；p. L755A（*n*=1）；p. L755P（*n*=1）。p. A775_G776insYVMA突变亚型患者的ORR和DCR均高于其他*HER2*突变亚型患者（52.9% *vs* 26.7%, *χ*^2^=2.281, *P*=0.131; 94.1% *vs* 93.3%, *χ*^2^=0.008, *P*=0.927），差异无统计学意义。

### 2.5 *HER2*基因状态及突变亚型与PFS之间的关系

单因素分析结果显示，有无脑转移、有无维持化疗和*HER2*基因状态是影响PFS的重要因素，而PFS与年龄、性别、是否有吸烟史、是否寡转移、有无肝转移及铂的种类无关（表 2）。将有无肝转移、有无脑转移、有无维持化疗和*HER2*基因状态纳入多因素分析研究对象，结果显示PFS与有无肝转移、有无脑转移、有无维持化疗均无关，*HER2*突变是PFS的正性独立预后因素（*P*=0.038）（表 3、图 1）。对*HER2*突变亚型进行单因素分析结果显示，p. A775_G776insYVMA突变患者的中位PFS为8.2个月，其他突变亚型的PFS为7.2个月，二者PFS差异无统计学意义（*χ*^2^=0.557, *P*=0.455）（图 1）。

**1:**
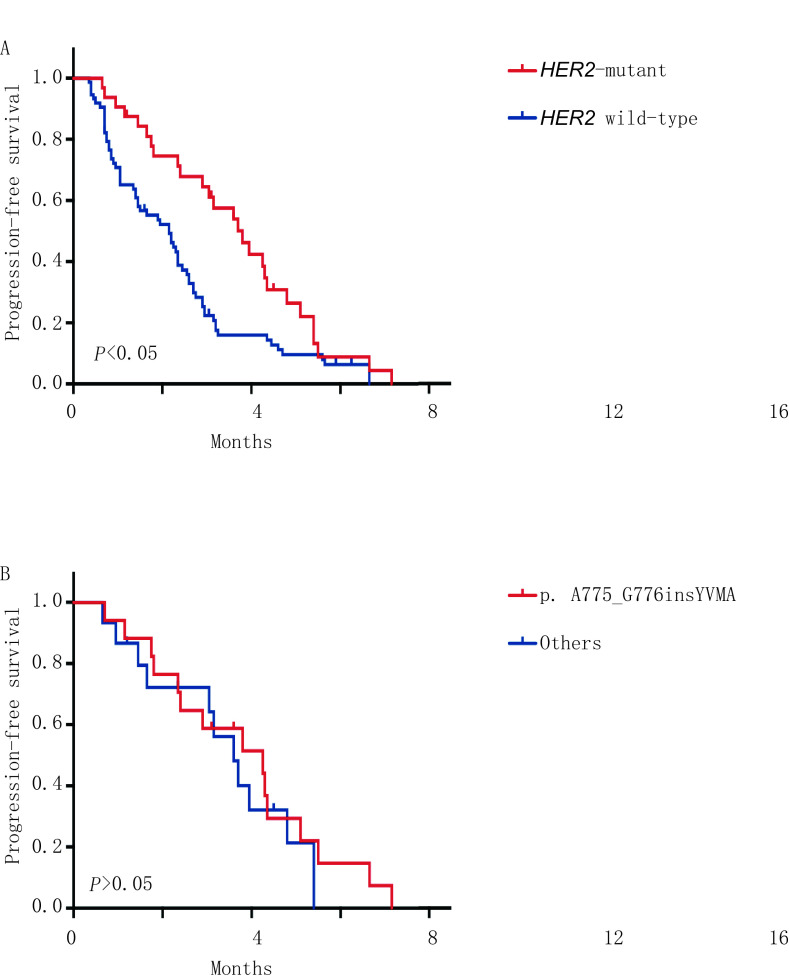
生存曲线。A：*HER2*突变与HER2野生型肺腺癌患者PFS曲线比较；B：p. A775_G776insYVMA与其他突变亚型PFS曲线比较。 Survival curve of the patients. A: Comparison of PFS in *HER2*-mutatant with HER2 wild-type lung adenocarcinoma patients; B: Comparison of PFS in the p. A775_G776insYVMA group with the other variants group. PFS: progression-free survival.

**2:** 106例患者临床特征与预后关系的单因素分析 Single factor analysis of the relationship between clinical features and prognosis-free survival (PFS) of 106 cases

Characteristic	*n*	Median PFS (mo)	*χ*^2^	*P*
Age (yr)			0.168	0.682
< 65	73	4.9		
≥65	33	3.9		
Gender			1.847	0.174
Male	63	3.9		
Female	43	5.8		
Smoking status			1.756	0.185
Ever	46	4.4		
Never	60	5.4		
Oligometastases, at diagnosis			0.065	0.799
Yes	22	4.7		
No	84	4.7		
Liver metastases, at diagnosis			2.108	0.146
Yes	6	1.6		
No	100	4.7		
Brain metastases, at diagnosis			7.342	0.007
Yes	15	3.0		
No	91	5.2		
Platinum			0.380	0.827
Cisplatin	29	4.3		
Carboplatin	25	5.9		
Nedaplatin	52	4.7		
Maintenance chemotherapy			5.268	0.022
Yes	35	6.1		
No	71	2.9		
*HER2* gene status			7.632	0.006
Mutant	32	7.6		
Wild-type	74	4.3		

**3:** 多因素生存分析 Multivariate survival analysis

Variable	B	SE	Wald	RR	95%CI	*P*
Liver metastases（yes *vs* no）	0.914	0.528	2.998	2.494	0.886-7.016	0.083
Brain metastases（yes *vs* no）	0.464	0.316	2.152	1.590	0.856-2.956	0.142
Maintenance chemotherapy (yes *vs* no）	-0.422	0.243	3.008	0.656	0.407-1.056	0.083
*HER2* gene status	-0.506	0.244	4.288	0.603	0.373-0.973	0.038

## 3 讨论

*HER2*突变在NSCLC中发生率为1%-4%，在EGFR、ALK、KRAS均为阴性的选择人群中*HER2*突变率为6%，多数*HER2*突变在20号外显子发生插入突变，最常见的突变亚型是p. A775_G776insYVMA突变，该突变亚型是在20号外显子的第776位密码子YVMA序列处重复或插入12个碱基对，导致PI3K-AKT和MEK-ERK通路的下游活化，从而促进细胞的增殖^[7-9]^。本研究中，*HER2*突变在EGFR、ALK、ROS-1、KRAS、BRAF、RET、MET均为阴性的患者中占30.2%，国内外尚未有研究报道*HER2*突变在上述7种基因均为阴性的患者中的发生率。考虑该研究属于小样本研究，可能存在抽样偏差等因素，这一结果有待未来的大样本、多中心研究进一步证实。*HER2*突变在本研究中显示出独特的临床特征，多见于年轻、不吸烟的女性患者，与之前研究^[6, 8-10]^报道相符。

虽然*HER2*突变未来可能成为NSCLC靶向治疗的新靶点，但近20年来的研究仍未确定疗效确切的抗HER2分子靶向治疗药物。美国国立综合癌症网络（National Comprehensive Cancer Network, NCCN）指南因为曲妥珠单抗和阿法替尼治疗缓解率低，已将二者从*HER2*突变NSCLC患者的治疗建议中删除^[11]^。拉帕替尼、来那替尼和达克替尼则在多项研究中显示出了更差的治疗效果^[12-15]^。近期有数据^[16, 17]^显示阿多曲妥珠单抗依酯（ado trastuzumab emtansine, T-DM1）和波齐替尼在*HER2*突变晚期NSCLC患者中有较好的治疗效果，TDM-1靶向治疗*HER2*突变患者的ORR为44%，中位PFS为5个月；波齐替尼的ORR为54.5%，中位PFS为6.2个月。但目前TDM-1和波齐替尼均未在中国上市，患者获得药物有一定难度，且一线使用TDM-1或波齐替尼或化疗孰优孰劣尚不明确，因此大多数*HER2*突变肺腺癌患者一线治疗以化疗为主，其中最常见的化疗方案为培美曲塞联合铂类。

Tsai等^[18]^通过Northern印记分析发现，*HER2*基因过表达的NSCLC与多种化疗药物的原发性耐药相关。之后国内一项研究^[19]^显示，肺腺癌培美曲塞的继发性耐药可能与HER2表达上调有关，耐药机制可能是通过增强DNA修复活性、促进细胞增殖和抗凋亡发挥作用。而*HER2*突变状态是否影响培美曲塞化疗的疗效尚不明确。2016年，Eng等^[20]^研究报道18例携带*HER2*突变的Ⅳ期肺腺癌患者应用含培美曲塞方案一线化疗的中位持续时间为8.8个月。与Eng等研究不同的是，本研究通过评估患者影像学的改变并根据RECIST 1.1进行疗效判定，排除了由于化疗无法耐受或患者主观意愿而中止化疗等影响，从而获得较为客观的疗效评价结果和PFS数据。Wang等^[6]^在晚期肺腺癌患者一线使用含培美曲塞化疗疗效的研究中发现，*HER2*突变患者的PFS与*KRAS*突变的PFS相似（5.1个月*vs* 5个月，*P* > 0.05），劣于ALK/ROS1重排患者（5.1个月*vs* 9.2个月，*P* < 0.05）和EGFR突变患者（5.1个月*vs* 6.5个月，*P* > 0.05）。本研究中，*HER2*突变患者的ORR和DCR分别为40.6%和93.8%，PFS为7.4个月，均优于Wang等^[6]^的研究结果，分析可能与Wang等^[6]^的研究中部分患者初始使用培美曲塞单药化疗，且多数患者未进行后续维持化疗相关。本研究也对*HER2*突变亚组间进行了分析，结果显示p. A775_G776insYVMA突变者的PFS优于其他突变亚型患者，与Wang等^[6]^研究报道不符，有待进一步研究探索。本研究评价远期化疗疗效时只对PFS数据进行了分析，未分析这一人群的总生存时间（overall survival, OS）数据，原因在于患者的二线及后续治疗情况各异，鉴于回顾性研究本身存在的局限性，*HER2*基因状态是否与接受一线培美曲塞联合铂类化疗患者的OS相关，需要进一步前瞻性研究进行深入探讨。

综上所述，在晚期肺腺癌患者的一线培美曲塞联合铂类化疗中，*HER2*突变患者的ORR、DCR和PFS均优于HER2野生型患者，提示相比HER2野生型肺腺癌患者，*HER2*突变患者一线应用培美曲塞联合铂类的化疗有更大的临床获益。
